# STEM-Gender Stereotypes: Associations With School Empowerment and School Engagement Among Italian and Nigerian Adolescents

**DOI:** 10.3389/fpsyg.2022.879178

**Published:** 2022-07-06

**Authors:** Pasquale Musso, Maria Beatrice Ligorio, Ebere Ibe, Susanna Annese, Cristina Semeraro, Rosalinda Cassibba

**Affiliations:** ^1^Department of Educational Sciences, Psychology, Communication, University of Studies of Bari Aldo Moro, Bari, Italy; ^2^Department of Science Education, University of Nigeria, Nsukka, Nigeria

**Keywords:** STEM-gender stereotypes, school empowerment, school engagement, socio-economic status, cultural comparison

## Abstract

While many sociocultural, contextual, biological, behavioral, and psychological variables may contribute to the widespread under-representation of girls and women in the science, technology, engineering, and mathematics (STEM) field, this study focused on STEM-gender stereotypes, school experiences, and adolescence as critical factors in driving students' interest and motivation in STEM. Based on this, the study (a) investigated differences by gender and national context (Italy vs. Nigeria) in adolescents' STEM-gender stereotypes, school empowerment, and school engagement in a preliminary step, and (b) simultaneously examined how adolescents' STEM-gender stereotypes were related to school empowerment and school engagement as well as to socioeconomic status (SES). These latter relations were considered within the context of the potential moderating role of gender and national context. Participants included 213 Italian adolescents (M_age_ = 13.91; 52.1% girls) and 214 Nigerian adolescents (M_age_ = 13.92; 60.3% girls), who completed measures of school empowerment and engagement, STEM-gender stereotypes, and SES. A multivariate analysis of covariance showed that Nigerian girls and boys reported significantly higher levels of school empowerment, school engagement, and STEM-gender stereotypes than their Italian peers. Moreover, regardless of the national context, boys scored significantly higher on school empowerment and STEM-gender stereotypes than girls. Furthermore, a multiple-group path analysis revealed how higher school empowerment was related to lower STEM-gender stereotypes in both Italian and Nigerian girls' groups, while higher school engagement was associated with lower STEM-gender stereotypes only in the Nigerian groups. Regardless of gender and nationality, higher SES was linked to lower STEM-gender stereotypes. These findings particularly suggest that school empowerment and school engagement can be relevant dimensions to be studied and to develop strategies to counteract STEM-gender stereotypes in adolescence. Nonetheless, gender and national context are key factors to be considered. Limitations, strengths, future research, and educational implications are discussed.

## Introduction

Science, technology, engineering, and mathematics (STEM) education is one of the key factors for preparing students for in-demand careers worldwide (e.g., Marginson et al., 2013; Zilberman and Ice, 2021; Eurostat, 2022). The continually evolving STEM sectors produce increasing opportunities to find entry-level work positions. This trend is proven not only in low- and middle-income countries, like those in Africa, where STEM education is strongly supported as a critical investment for social and economic development (World Bank, 2014) but also in high-income industrial countries. For example, in Italy 80% of STEM graduates find work within 1 year after graduation and this percentage becomes 92.1% within 5 years after graduation. These employment rates are significantly higher than those observed for graduates as a whole (AlmaLaurea, 2022) and confirm that new jobs are emerging within our economies, which require knowledge and skills in STEM. In addition to youth employment issues, global problems such as climate change, nutrition of a growing population, or growth of the economy itself can be better afforded by a new generation of well-educated young people in STEM. Therefore, STEM education has become a priority issue for both researchers from different fields and policymakers and non-governmental organizations.

However, despite this push toward the multiplication of actions to favor the spread of STEM education and employment, many countries are facing increasing gaps in this field (Kramer et al., 2015). As the United Nations Children's Fund (UNICEF) recently pointed out (2020a) in a specific report—much of this gap depends also on the under-representation of girls and women in STEM. The UNICEF report maps gender equity in STEM in 86 countries in different areas of the world and shows remarkable results. First, in more than 60% of the countries, girls at school (both at the upper primary and secondary level) show “minimum levels of proficiency” (MLP) in math and science at least comparable or higher than boys, but substantial differences exist depending on the regional area and socioeconomic status (SES). For example, girls present significantly lower MLP in math in most developing countries in Sub-Saharan Africa and Latin America, while within countries, girls show lower MLP in math than boys in the context of lower (but not higher) SES. Second, considering the “high proficiency levels” (HPL) in STEM, girls are less likely than boys to achieve these levels in most of the countries: 72% for math and 56% for science in upper primary school and 96% and 83%, respectively, at secondary school. Third, in around 60% of the countries, girls have a significant lower level of self-confidence in their STEM abilities than boys starting from the upper primary school; in the other 40% of countries, self-confidence scores also tend to be lower for girls, although in a statistically non-significant way. Fourth, girls' lower self-confidence is linked to a gender gap in STEM engagement, interest, and enjoyment, with correlations ranging from 0.44 to 0.65. Fifth, in 92% of countries, more boys than girls aspire to a STEM career; this gender gap is also evident even in the groups with the highest levels of STEM proficiency, with more than a fifth of boys aspiring to a STEM career in 64% of the countries, while this percentage drops to 17% for girls.

What emerges from this set of findings is that the under-representation of girls and women in the STEM field is generally widespread. This situation has evident negative consequences not only in terms of the development of the individual potential of half of the world's population (i.e., creativity, innovation, problem-solving, or increasing work-related STEM skills), but also from a more general, social, and political point of view. Without equal access and participation in STEM, for example, the 2030 Agenda for Sustainable Development provided by the United Nations (2015) will hardly reach its goals. STEM for girls, in fact, can stimulate and accelerate a number of Sustainable Development Goals (SDGs), like gender equality (SDG 5), no poverty (SDG 1), good health and wellbeing (SDG 3), and decent work and economic growth (SDG 8). This is because through better knowledge and use of science and technology (for example, related to health or communications) girls and women can potentially improve their lives and work-related opportunities. This acceleration may also concern quality education (SDG 4) or industry, innovation, and infrastructure (SDG 9), because higher STEM abilities empower girls to contribute to developing transferable, technical, and vocational skills for entrepreneurship and to lead innovative solutions in industrial sectors (UNICEF, 2020b).

In view of such a context and the potential negative future scenarios that arise from it, it is extremely important to understand why girls are under-represented in STEM and what actions can be taken to reverse the trend. While many sociocultural, contextual, biological, behavioral, and psychological factors may contribute to limiting girls' engagement with STEM, Master and Meltzoff (2016) highlighted the critical contribution of gender stereotypes in driving young students' interest and motivation in STEM. The under-representation of girls and women in the STEM field is deeply rooted in gender social representations that suggest how girls are not appropriate, or at least less than boys, for STEM education and employment (Master et al., 2014; Piatek-Jimenez et al., 2018; Thébaud and Charles, 2018; UNICEF, 2020b). Data from the above-mentioned report from UNICEF (2020a) support this view by associating gender gap in STEM with a variety of gender norms, biases, and stereotypes (e.g., girls receive less STEM-related praise; parents expect their sons, rather than their daughters, to have a STEM career). Regarding gender stereotypes, in many of the countries included in the report, 70% of individuals considers STEM as adequate for males than for females (e.g., Nosek et al., 2009; Campos et al., 2014; Cheryan et al., 2015; Grunspan et al., 2016; UNESCO, 2017; Schleicher, 2019).

Focusing on gender stereotypes is consistent with the most recent evidence claiming that the most important explanations of gender differences are grounded in preferences and choices rather than in skills and performance (e.g., Riegle-Crumb et al., 2012; Dasgupta and Stout, 2014). This approach explains why there would be fewer or no reliable gender differences in primary school than at later school levels when girls and boys more actively express preferences and interests and have been exposed to gender stereotypes influence for a longer time. In their work on these issues, Master and Meltzoff (2020) provide at least two key suggestions. First of all, they distinguish between two dimensions of STEM-gender stereotypes (see also Master and Meltzoff, 2016; Wynn and Correll, 2017): a “cultural fit” stereotype (i.e., the belief that “STEM = male” and “girls like STEM less than boys”) and an “ability” stereotype (i.e., the belief that “girls have less ability than boys”). Girls and women may worry about not fitting into the image of a STEM person and not having the ability to succeed in STEM and this combination contributes to their STEM under-representation. This broadens the concept of stereotype threat (Steele, 1997) and the related research approach, usually focused on how ability stereotypes affect girls' and women's performance in STEM and suggests using appropriate measures to grasp simultaneously “cultural fit” and “ability” stereotypes. Also, they propose a comprehensive STEreotypes, Motivation, and Outcomes (STEMO) developmental model, in which social factors (e.g., stereotypes) are essential in explaining youth's interest and academic outcomes in STEM. Specifically, this model indicates that when individuals encounter stereotypes about social groups (e.g., STEM-gender cultural fit and ability stereotypes) and these stereotypes are relevant to their social identity (e.g., gender), this has an impact on their self-representations (i.e., identification, ability beliefs, and sense of belonging) in STEM and, consequently, compromise their interest and academic achievement (e.g., participation) in STEM.

The STEMO model is a promising avenue for future interventions, given the centrality of STEM-gender stereotypes and their potential malleability in the school settings. From this point of view, one of the possible interventions is to challenge stereotypes about who belongs to STEM (cultural fit stereotypes) and the possession of fixed abilities determined by gender (ability stereotypes). According to the STEMO model, such interventions would have the consequence of changing the way girls would see themselves, increasing aspects such as the sense of identification with the STEM domain (“I am a math person”), the self-efficacy (“I am able to be successful in science and technology”), and the sense of belonging (“I am part of the STEM group”). This would lead to more positive STEM outcomes. Thus, one of the central questions is to evaluate which contextual, individual, social, and cultural factors favor overcoming the “traditional” STEM-gender stereotypes. In this study, we addressed the issue by focusing on (a) school context and adolescence; (b) two individual factors related to school experience, namely school empowerment and school engagement, theoretically associated with STEM-gender stereotypes; (c) one social factor like SES, given its influence on STEM outcomes (see above); and (d) gender and cultural differences, by comparing girls and boys from a high-income industrial country, such as Italy, with girls and boys from a low-middle-income country, such as Nigeria.

We considered that the processes suggested by the STEMO model unfold with the experiences in school (Master and Meltzoff, 2020), which represents one of the primary socialization environments for children and youth in terms of STEM subjects and expectations. Teachers and school staff may hold STEM-gender stereotypes influencing their interactions with students (e.g., Gunderson et al., 2012) as well as students' STEM-gender stereotypes and self-concepts (e.g., del Río et al., 2019). At higher school levels, students organize their tertiary educational and career preferences and choices also based on these experiences. Therefore, school is a privileged context to be considered both in terms of understanding the mechanisms that boost or buffer the transmission of STEM-gender stereotypes and in terms of potential interventions. In addition, adolescence represents a crucial life phase to be considered in relation to STEM-gender stereotypes. In fact, the most recent literature has adequately supported that traditional STEM-gender stereotypes were more prevalent among adolescents compared to younger children (e.g., Passolunghi et al., 2014; Miller et al., 2018; Starr and Simpkins, 2021). This finding was explained through the peculiarities of adolescence, a period when individuals are engaged in identity formation and try to use more systematically the information deriving from social confrontation (Erikson, 1968). Stereotypes may contribute to the development of identity because adolescents have the cognitive abilities to relate stereotypes to themselves (e.g., Marcia, 1994; Patterson and Bigler, 2018). Hence, adolescents represent a crucial group to be studied within the STEM-gender stereotypes research context.

Given the importance of the school context, dimensions such as school empowerment (Tam et al., 2020; Ruiz-Cantisani et al., 2021) and school engagement (Almeda and Baker, 2020) can play a role in the formation of STEM-gender stereotypes and STEM gender gap. Previous research suggested how there are links between STEM-gender stereotypes and self-efficacy: girls or women with higher explicit or implicit gender stereotypes in a STEM domain (e.g., math or science) frequently show lower beliefs to succeed in such a domain (e.g., Deemer et al., 2014; Passolunghi et al., 2014; Ertl et al., 2017). According to Bandura (1982), self-efficacy is subject-specific, and it should be conceptualized separately in each STEM domain. However, Zimmerman and Warschausky (1998) highlighted how self-efficacy is only a component of psychological empowerment, which “is not simply the belief that an individual can overcome barriers to independence, but also includes the individual's capacity and willingness to make such an effort (p. 13).” Cattaneo and Chapman (2010) argued that empowerment focuses on personally meaningful goals and aims to enhance one's social influence to exert power in social interaction. Starting from these conceptualizations, we assumed empowerment as a process that helps people gain control over their own lives (Page and Czuba, 1999), including reduced effects of stereotypes held by a society or community. The school community plays a relevant role in providing opportunities to experience psychological empowerment. Thus, higher levels of psychological empowerment experienced in the school context (school empowerment) could be related to lower levels of STEM-gender stereotypes, which usually reduce power in social influence and life choices. Furthermore, considering the STEM gender gap and the contents of STEM-gender stereotypes, it is still possible to assume a (negative) relation between school empowerment and STEM-gender stereotypes in girls, but not in boys.

The research also showed how students' engagement in the school context (school engagement) may be associated with STEM aspirations (Cunningham et al., 2015) and with the type of school programs chosen by the students, with those in STEM programs more highly engaged than those in traditional programs (Patel et al., 2013; Kogo-Masila, 2017). However, the literature examining the association of school engagement with STEM-related dimensions is limited. To the best of our knowledge, no studies have investigated the relation between school engagement and STEM-gender stereotypes. Despite this paucity, there are reasons for this link to be explored. School engagement may be conceptualized as active, goal-directed, constructive interactions with the physical, social, and cultural environments of school (Furrer and Skinner, 2002) and, at a more individual level, may be operationalized as energy (i.e., positive approach), dedication (i.e., positive cognitive attitude), and absorption (i.e., concentration abilities) directed to school activities (Salmela-Aro and Upadyaya, 2012). Students who feel engaged with school show higher motivation and academic achievement over time (Salmela-Aro and Upadyaya, 2012); for girls, this dynamic may trigger greater curiosity and interest in STEM subjects as well as STEM-gender stereotype reactance with increased effort and willingness to demonstrate that the stereotypes are biased. Furthermore, this potential process can be more easily detectable in national contexts where the school still represents a concrete means for social redemption and where therefore school engagement can have more relevant outcomes from this point of view (i.e., more in a low-middle-income country rather than in a high-income country).

Social factors are also involved in students' STEM-gender stereotypes. Students from higher SES backgrounds may have previously been given more opportunities to learn about STEM and to build their STEM skills. This is especially important for girls, who can maximize their potential for success in STEM and, consequently, construct less biased STEM-gender stereotypes (Master and Meltzoff, 2020). On the contrary, girls from lower-SES backgrounds have fewer learning opportunities in STEM and chances to experience STEM skills; therefore, they may be more easily adherent to the culturally transmitted STEM-gender stereotypes.

Both STEM-gender stereotypes and the individual and social factors just described as well as their relations may vary depending on the students' gender and the national context of reference. STEM-gender stereotypes are cultural representations expressed by a particular society in many ways, such as social interactions and language use (Markus and Kitayama, 2010; Master and Meltzoff, 2020). They transcend beliefs within an individual, but when stereotypes concern issues involving gender, they can favor a gender more than another. Traditional STEM-gender stereotypes favor the boys, who may tend to conform to them less critically and present higher levels of stereotypes than girls, especially during adolescence (see Starr and Simpkins, 2021). Also, in low- and middle-income economies with higher levels of gender gap, STEM-gender stereotypes may be more prevalent (UNICEF, 2020b; World Economic Forum, 2021) than in developed countries, where all genders grow up by believing they share the same opportunities. Furthermore, previous studies reported consistent gender differences in school engagement, with girls more engaged with school than boys (e.g., Wang and Eccles, 2012; Fernández-Zabala et al., 2016). As Wang and Eccles (2012) reported, this finding may reflect a greater girls' concern for school performance, maybe because of gender socialization processes and differential expectations of parents and teachers (see also Wilkinson and Marrett, 1985; Eccles, 2007). The research also highlighted gender differences for school empowerment, with females scoring higher than males, and justified such a finding with the relevance of the social dimension for girls compared to boys (Helgeson, 1994; Årdal et al., 2018); yet these differences are small and further studies on this topic are needed. As for the differences related to the national context, to the best of our knowledge, literature does not report how school engagement and empowerment may change depending on country income levels (low and middle vs. high). However, it is theoretically possible that when school represents a greater opportunity for social mobility (in low- and middle-income countries), school engagement may be higher. Also, in terms of the relations among STEM-gender stereotypes, school empowerment, and school engagement, the research seems to be specifically lacking. Nevertheless, starting from the related literature, we previously suggested that (a) higher school empowerment may be associated with lower STEM-gender stereotypes in girls and (b) higher school engagement may be more associated with lower STEM-gender stereotypes in low-middle-income countries than in high-income countries, especially for girls. Finally, regarding the link between SES and STEM-gender stereotypes, previous research suggested that higher SES is associated with lower levels of STEM-gender stereotypes, and this is particularly evident for girls (Master and Meltzoff, 2020).

### Aims and Hypotheses

In light of previous arguments, this study addressed the following two aims: (a) to assess gender and cultural differences in adolescents' STEM-gender stereotypes, school empowerment, and school engagement as a preliminary step; and (b) to analyze the associations of adolescents' school empowerment, school engagement, and SES with STEM-gender stereotypes and how these relations may change depending on gender and cultural context. To achieve these goals, as previously mentioned, we referred to two specific national contexts such as Italy and Nigeria, which are interesting to compare due to their socioeconomic and cultural characteristics. Italy is a European westernized country and one of the world's most industrialized economy with high-income levels. The gross domestic product (GDP) in Italy was 1,890 billion US dollars in 2020, according to official data from the World Bank (2022a). However, despite an improvement in the global gender gap index during the last 15 years, Italy is in the 63rd place across the 156 countries covered by the 2021 Global Gender Gap Report (GGGP, World Economic Forum, 2021) and presents a ratio of 1:0.46 in terms of STEM attainment in favor of males. Nigeria is a low-middle- income country, located in the western Sub-Saharan Africa. The GDP in Nigeria was 432 billion US dollars in 2020 (World Bank, 2022b). Nigeria experienced a slight improvement in the global gender gap index during the last 15 years as well, but it ranks 139th among the 156 countries (World Economic Forum, 2021). Although the 2021 GGGP does not report any indications about the male-female ratio of STEM attainment, a number of reports have highlighted how Sub-Saharan Africa has one of the largest gender gaps worldwide in STEM, especially in the lower secondary school (e.g., Rubiano-Matulevich et al., 2019), and Nigeria presents a very low participation of females in STEM courses as a result of cultural and religious beliefs, traditions, early marriage, and parental educational background (e.g., Salman et al., 2011; Abdullahi et al., 2019).

Based on all the above information, we predicted that:

a) STEM-gender stereotypes were higher for boys than girls and for the Nigerian than the Italian adolescents.
b) School engagement was higher for girls than boys and in the Nigerian than in the Italian adolescents.
c) Higher school empowerment was significantly associated with lower STEM-gender stereotypes for girls, but not for boys.
d) Higher school engagement was more significantly associated with lower STEM-gender stereotypes in the Nigerian than in the Italian adolescents, especially for girls.
e) Higher SES was associated with lower levels of STEM-gender stereotypes, more significantly for girls than boys.

Given the lacking or less consistent literature as well as the exploratory nature of the study, we did not predict any specific gender and cultural differences for mean levels of school empowerment.

## Method

### Participants

The participants in this study included 213 Italian adolescents (*M*_*age*_ = 13.91, *SD* = 0.38, range = from 12 to 15 years; 47.9% boys and 52.1% girls) and 214 Nigerian adolescents (*M*_*age*_ = 13.92, *SD* = 0.97, range = from 12 to 15 years; 39.7% boys and 60.3% girls). Both the Italian and Nigerian participants attended the last year of lower secondary school; therefore, the following year, they would choose the higher education path that would lead them to a more restricted career perspective. This, therefore, represented a pivotal phase in their social identity development and sensitivity to STEM-gender stereotypes (see Introduction section). The Italian adolescents were attending school in southerneastern Italy (Apulia region) and Nigerian adolescents in southeastern Nigeria (Enugu State) in towns with more than 100,000 inhabitants. The average number of students in the classes frequented by the participants was 21.67 (*SD* = 3.59) for the Italian group and 38.19 (*SD* = 4.68) for the Nigerian group. The SES of the participants' families was prevalently medium. Based on a three-level classification of scores using the Barratt Simplified Measure of Social Status (BSMSS, Barratt, 2012, see Measures section), 4.2% of Italian and 9.3% of Nigerian adolescents fell into the low stratum, 62.0% of Italian and 58.9% of Nigerian adolescents fell into the medium stratum, and 33.8% of Italian and 31.8% of Nigerian adolescents fell into the high stratum. A comparison of the two national groups showed that they did not differ significantly in terms of gender (0 = boys, 1 = girls), χ^2^(1) = 2.89, *p* = 0.09, SES (0 = low, 1 = medium, 2 = high), χ^2^(2) = 4.42, *p* = 0.11, and age, *t*(425) = −0.17, *p* = 0.87. Significant differences were found for the average number of students in the classes, *t*(425) = −40.98, *p* < 0.001, with the Nigerian school classes more numerous than the Italian ones.

### Procedure

The study was approved on 11 May 2020, by the Ethical Committee at the Department of Education, Psychology, and Communication at the University of Bari (Ethics reference code: ET-20-06), and all procedures were performed following the ethical principles for psychological research of the Italian Association of Psychology (2015). A convenience sample was initially recruited from three schools in the Italian urban context in Italy. The schools were selected by internal University search databases, where a list of local school institutions was stored, and encouraged to take part in the investigation through a motivation letter introducing the purpose of the research work. Within 1 month, the same procedure was followed in Nigeria by the third author of this work, who also ensured the comparability of the Nigerian schools with the Italian ones through a specific pairing process, by considering the schools' regional location in Nigeria and the urban characteristics in which they were inserted. After receiving permission from the respective school principals, the students' parents from both Italy and Nigeria were informed through a letter describing the purposes of the research, the voluntary nature of participation, and the anonymity of responses. All the parents provided informed consent for their son's or daughter's participation. In addition, participants provided signed assent agreeing to take part in the study. Participants completed a web-based survey in Italy and a web-based or a paper-and-pencil survey in Nigeria (depending on the schools) during the class time and they could withdraw at any time. The data collection took place between April and June 2021. Usually, participants completed the survey in about 30 min.

### Measures

The measures used in this study were presented in the Italian language for the Italian participants, and in English for the Nigerian participants as it is the official and widely used language in Nigeria. When it was the case, we translated some measures from English into Italian (i.e., school engagement inventory). In the latter case, following the recommendations of the International Test Commission (2017), an independent English native language teacher, fluent in Italian, did a back-translation. Slight discrepancies were resolved through discussion and consensual agreement.

#### Socio-Demographics

Respondents were asked to indicate their age and gender. Paternal and/or maternal level of school completed (scores from 3 = *less than 7th grade* to 21 = *graduate degree*) and the parents' occupation (scores from 5 = *e.g., day laborer, house cleaner, food preparation worker* to 45 = *e.g., physician, judge, senior manager*) were assessed using BSMSS (Barratt, 2012; total education + total occupation scores from 8 to 66).

#### School Empowerment

An adapted form of the Psychological Empowerment Scale (PES; Spreitzer, 1995; see Pietrantoni and Prati, 2008, for the Italian version) was used to assess students' perception of school empowerment. The original version of the PES consists of 12 items assessing four different dimensions in the workplace comprising three items each: meaning, competence, self-determination, and impact. However, recently it was adapted among students in different national contexts (e.g., Beauvais et al., 2014; Azizi et al., 2020; Cayaban et al., 2022). Following this line, we culturally adapted the instrument to students and the school environment. In doing so, the first three authors worked together following a specific procedure (see, for example, da Silva Augusto et al., 2017). Preliminarily, they discussed conceptual and semantic characteristics of PES, as previously adapted in the academic context, in light of the idiomatic and cultural differences (or equivalences) between the English and Italian versions as well as the Nigerian and Italian contexts. They agreed on the need to assess cross-culturally the PES content validity, which is the degree to which each item was relevant to and representative of school empowerment. Thus, they recruited a committee of six experts (three Italian and three Nigerian) with extensive experience in the school context, who rated each item on a Likert-type scale from 1 (*not important*) to 4 (*very important*). Only the items that obtained the maximum score (i.e., 4) from at least two Italian and two Nigerian experts were considered valid. Five items met this criterion, with at least one item in one of the four initial dimensions of the PES. After excluding (to maintain the item-dimension balance) the item with less agreement among experts, the final scale had four items: “The study I do is very important to me” for meaning, “I am confident about my ability to study” for competence, “I have opportunity for independence and freedom in how I study” for self-determination, and “I have significant influence over what happens in my class” for impact. Items were scored by the participants on a Likert-type scale ranging from 1 (*strongly disagree*) to 5 (*strongly agree*). Prior studies have provided evidence that PES items load on four factors corresponding to the theoretical dimensions and that these factors load on a second-order factor of empowerment (e.g., Spreitzer, 1995; Pietrantoni and Prati, 2008). Thus, we expected that our four selected items would load on one factor of school empowerment across the two national contexts. We tested this one-factor structure model, as well as measurement invariance (configural, metric, and scalar, see Van de Schoot et al., 2012) across contexts, through robust maximum likelihood multi-group confirmatory factor analysis (MG-CFA; see the “Analysis Plan” section for model fit criteria), using the four items as observed indicators. This one-factor and scalar measurement-invariant model was adequately supported, χ^2^(10) = 16.33, *p* = 0.09, CFI = 0.970, RMSEA = 0.054, SRMR = 0.098. The internal consistency reliability scores calculated by the factor determinacy (Muthén and Muthén, 2012) were good for both the Italian (0.84) and Nigerian (0.78) groups. Overall, these results allowed validly comparing scale mean scores across the two national contexts (e.g., van de Vijver and Leung, 1997; Boer et al., 2018). For both groups, a composite variable was created by computing the average of the items, with higher scores indicating higher levels of school empowerment.

#### School Engagement

The Schoolwork Engagement Inventory (SEI; Salmela-Aro and Upadyaya, 2012) was used to assess students' perception of school engagement. The SEI consists of nine items assessing three different dimensions comprising three items each: energy, dedication, and absorption. To culturally adapt the instrument, we followed a procedure very similar to that already described for the school empowerment. The final scale had three items: “I feel strong and vigorous when I am studying” for energy, “I am enthusiastic about my studies.” for dedication, and “Time flies when I am studying” for absorption. The items were scored by the participants on a Likert-type scale ranging from 1 (a *couple of times a year*) to 5 (*daily*). Prior studies have provided evidence that SEI items load better on one factor among the younger students (e.g., Salmela-Aro and Upadyaya, 2012). Following this line, we expected that our three selected items would load on one factor of school engagement across the two national contexts. We tested this one-factor structure model, as well as measurement invariance across contexts, through MG-CFA, using the three items as observed indicators. This one-factor and scalar measurement-invariant model was adequately supported, χ^2^(4) = 7.75, *p* = 0.10, CFI = 0.975, RMSEA = 0.066, SRMR = 0.085. The factor determinacy scores were good for both the Italian (0.94) and Nigerian (0.78) groups. For both groups, a composite variable was created by computing the average of the items, with higher scores indicating higher levels of school engagement.

#### STEM-Gender Stereotypes

To assess STEM-gender stereotypes, we used an eight-item questionnaire adapted by Tomasetto et al. (2015). This questionnaire measures explicit stereotypes concerning both between-gender (i.e., “I believe that generally males are more talented than females at math/science-technology”) and within-gender (i.e., “I believe that generally females have more facility with language than with math/science-technology”) differences in math (four items) and science-technology (four items). As it is possible to understand from the example items, the between-gender stereotypes recall the “ability” stereotypes, while the within-gender stereotypes recall the “cultural fit” stereotypes proposed by the STEMO model (Master and Meltzoff, 2020). To culturally adapt the instrument, we followed a procedure similar to that already described for the previous measures. All the items were retained. They were scored on a Likert-type scale ranging from 1 (*strongly disagree*) to 5 (*strongly agree*). Prior studies have provided evidence that math-gender stereotypes items load on one factor (e.g., Tomasetto et al., 2015). Following this line, we expected that our eight items would load on two factor of math-gender and science/technology-gender stereotypes across the two national contexts. We tested this two-factor structure model, as well as measurement invariance across contexts, through MG-CFA, using the eight items as observed indicators. This two-factor and scalar measurement-invariant model was sufficiently supported, χ^2^(46) = 107.71, *p* < 0.001, CFI = 0.946, RMSEA = 0.079, SRMR = 0.094. The factor determinacy scores were good for both the Italian (0.96 and 0.97, respectively, for math-gender and science/technology-gender stereotypes) and Nigerian (0.92 and 0.94, respectively, for math-gender and science/technology-gender stereotypes) groups. However, the correlation between the two factors was very high: 0.95 for the Italian group and 0.78 for the Nigerian group. Based also on subsequent key analyses suggesting no differences in the patterns of results when considering math-gender and science/technology-gender stereotypes separately or as a whole, we used a unique variable of STEM-gender stereotypes henceforth for parsimony. The Cronbach's alpha coefficients for this general variable were: 0.96 for the Italian group and 0.85 for the Nigerian group. For both groups, a composite variable was created by computing the average of the eight items, with higher scores indicating higher levels of STEM-gender stereotypes.

### Analytic Plan

The data analysis proceeded in three main steps. First, descriptive statistics for the study variables were initially calculated using version 24 of the *Statistical Package for the Social Sciences* (SPSS). Specifically, mean scores, standard deviations, normality statistics, and bivariate correlations were computed.

Second, we evaluated differences by gender (0 = boys; 1 = girls) and national context (0 = Italy; 1 = Nigeria) in school empowerment, school engagement, and STEM-gender stereotypes. Particularly, we conducted a multivariate analysis of covariance (MANCOVA) considering gender and national context as independent variables and the other constructs as dependent variables. SES was entered as a covariate.

Third, to explore the differential associations of school empowerment, school engagement, and SES with STEM-gender stereotypes and how these relations varied by gender and national context, a multiple-group path analysis using *Mplus 7* (Muthén and Muthén, 2012) was performed considering four groups: Italian boys, Italian girls, Nigerian boys, and Nigerian girls. We initially estimated and compared an unconstrained (less restrictive) model, in which the most relevant path coefficients were allowed to vary between the four groups, with a constrained (more restrictive) model, where all key path coefficients were set equal across groups. Significant differences in fit between these models implied the estimation of alternative partially constrained models. We relied on well-known goodness-of-fit indices and their associated cutoffs to evaluate the model fit (e.g., Kline, 2015): chi-square (χ^2^) test with *p* > 0.05, CFI ≥ 0.90, RMSEA ≤ 0.08, and SRMR ≤ 0.10. To ascertain significant differences between nested models (the more vs. less restrictive model), at least two of these four criteria had to be satisfied (Kline, 2015): Δχ^2^ significant at *p* < 0.05, ΔCFI ≤ −0.010, ΔRMSEA ≥ 0.015, and ΔSRMR ≥ 0.010.

## Results

### Preliminary Analyses

An initial data screening revealed that three participants (two Italians and one Nigerian) did not complete the survey (more than 30% of responses were not completed). These cases were deleted from the dataset. Tables 1–3 summarize the descriptive statistics and report bivariate correlations in the total sample and by gender, by national context, and by gender and national context. They show how some observed variables were only slightly not normally distributed with skewness and kurtosis values > ± 1.00 (Kline, 2015). This permitted us to perform the MANCOVA with some confidence, while in the structural equation modeling environment, the data were however analyzed using robust maximum likelihood estimation methods.

**Table 1:** Means, standard deviations, skewness, and kurtosis for the key study variables for the entire sample, by gender, by national context and by gender and national context.

		***M***	***SD***	**Skewness**	**Kurtosis**
Entire sample (*N* = 427)					
1.	School empowerment (scored 1–5)	3.85	0.71	−0.34	−0.08
2.	School engagement (scored 1–5)	3.84	1.25	−0.96	−0.22
3.	STEM-gender stereotypes (scored 1–5)	2.83	1.19	0.00	−0.83
4.	Socio-economic status (scored 8–66)	48.42	14.29	−0.38	−0.92
Male group (*n* = 187)					
1.	School empowerment (scored 1–5)	3.93	0.71	−0.47	0.33
2.	School engagement (scored 1–5)	3.80	1.25	−0.92	−0.32
3.	STEM-gender stereotypes (scored 1–5)	3.00	1.27	−0.12	−0.95
4.	Socio-economic status (scored 8–66)	47.55	15.58	−0.41	−0.93
Female group (*n* = 240)					
1.	School empowerment (scored 1–5)	3.79	0.71	−0.26	−0.30
2.	School engagement (scored 1–5)	3.86	1.25	−0.99	−0.12
3.	STEM-gender stereotypes (scored 1–5)	2.70	1.12	0.04	−0.69
4.	Socio-economic status (scored 8–66)	49.11	13.20	−0.27	−1.13
Italian group (*n* = 213)					
1.	School empowerment (scored 1–5)	3.63	0.72	−0.37	0.03
2.	School engagement (scored 1–5)	3.31	1.32	−0.43	−1.09
3.	STEM-gender stereotypes (scored 1–5)	2.16	1.02	0.40	−0.62
4.	Socio-economic status (scored 8–66)	48.74	14.09	−0.41	−0.90
Nigerian group (*n* = 214)					
1.	School empowerment (scored 1–5)	4.07	0.64	−0.18	−0.78
2.	School engagement (scored 1–5)	4.36	0.91	−1.74	2.87
3.	STEM-gender stereotypes (scored 1–5)	3.50	0.95	−0.16	−0.48
4.	Socio-economic status (scored 8–66)	48.11	14.52	−0.35	−0.93
Italian male group (*n* = 102)					
1.	School empowerment (scored 1–5)	3.73	0.70	−0.54	0.75
2.	School engagement (scored 1–5)	3.40	1.34	−0.50	−1.11
3.	STEM-gender stereotypes (scored 1–5)	2.36	1.08	0.22	−0.63
4.	Socio-economic status (scored 8–66)	48.94	15.16	−0.61	−0.59
Italian female group (*n* = 111)					
1.	School empowerment (scored 1–5)	3.54	0.72	−0.23	−0.37
2.	School engagement (scored 1–5)	3.23	1.29	−0.39	−1.04
3.	STEM-gender stereotypes (scored 1–5)	1.97	0.93	0.48	−0.74
4.	Socio-economic status (scored 8–66)	48.55	13.10	−0.13	−1.46
Nigerian male group (*n* = 85)					
1.	School empowerment (scored 1–5)	4.17	0.64	−0.35	−0.64
2.	School engagement (scored 1–5)	4.29	0.91	−1.48	1.87
3.	STEM-gender stereotypes (scored 1–5)	3.75	1.04	−0.74	0.05
4.	Socio-economic status (scored 8–66)	45.87	16.00	−0.18	−1.15
Nigerian female group (*n* = 129)					
1.	School empowerment (scored 1–5)	4.00	0.63	−0.08	−0.78
2.	School engagement (scored 1–5)	4.40	0.91	−1.94	3.79
3.	STEM-gender stereotypes (scored 1–5)	3.34	0.85	0.21	−0.37
4.	Socio-economic status (scored 8–66)	49.59	13.31	−0.40	−0.83

**Table 2:** Pearson's bivariate correlations for the Italian sample.

		**1**.	**2**.	**3**.	**4**.
1.	School empowerment (scored 1–5)		0.48***	−0.14	0.18
2.	School engagement (scored 1–5)	0.51***		−0.02	0.01
3.	STEM-gender stereotypes (scored 1–5)	0.27**	0.12		−0.09
4.	Socio-economic status (scored 8–66)	0.18	0.09	−0.19	

*Upper diagonal: correlation matrix for females (n = 111). Lower diagonal: correlation matrix for males (n = 102).*

** *p < 0.01,*

*** *p < 0.001*.

**Table 3:** Pearson's bivariate correlations for the Nigerian sample.

		**1**.	**2**.	**3**.	**4**.
1.	School empowerment (scored 1–5)		−0.01	−0.18*	0.21*
2.	School engagement (scored 1–5)	0.12		−0.24**	−0.09
3.	STEM-gender stereotypes (scored 1–5)	0.00	−0.20		−0.14
4.	Socio-economic status (scored 8–66)	0.26*	0.05	−0.03	

*Upper diagonal: correlation matrix for females (n = 129). Lower diagonal: correlation matrix for males (n = 85).*

* *p < 0.05*,

** *p < 0.01*.

### Mancova

Results from the MANCOVA showed a significant multivariate effect of gender, Wilks' Lambda = 0.95, *F*_(3,420)_ = 7.94, *p* < 0.001, η^2^ = 0.05, and national context, Wilks' Lambda = 0.57, *F*_(3,420)_ = 106.99, *p* < 0.001, η^2^ = 0.43. Two-way effects were not statistically significant. Follow-up univariate analyses (see Table 4) indicated that school empowerment and STEM-gender stereotypes differed significantly across gender, as well as school empowerment, school engagement, and STEM-gender stereotypes differed significantly across national contexts. Specifically, pairwise comparisons revealed that Nigerian participants reported significantly higher levels of all dependent variables than their Italian peers. Moreover, boys scored significantly higher on school empowerment and STEM-gender stereotypes than their female peers.

**Table 4:** Univariate analyses of covariance and pairwise comparisons for gender and national context (Italian vs. Nigerian) on school empowerment, school engagement, and STEM-gender stereotypes.

	**MANCOVA-adjusted means by gender**				**MANCOVA-adjusted means by national context**			
	**Male (*n* = 187)**	**Female (*n* = 240)**	***F*(1, 422)**	**η^2^**	**Italian (*n* = 213)**	**Nigerian (*n* = 214)**	***F*(1, 422)**	**η^2^**
School empowerment	3.96^a^	3.76^b^	9.21**	0.02	3.63^a^	4.09^b^	50.66***	0.11
School engagement	3.84	3.82	0.06	0.00	3.31^a^	4.35^b^	86.83***	0.17
STEM-gender stereotypes	3.05^a^	2.66^b^	17.30***	0.04	2.17^a^	3.54^b^	210.88***	0.33

*A mean is significantly different (p < 0.05) from another mean within the same row if they have different superscripts (a or b).*

** *p < 0.01*,

*** *p < 0.001. MANCOVA, multivariate analysis of covariance*.

### Multiple-Group Path Analysis

**Figure 1:**
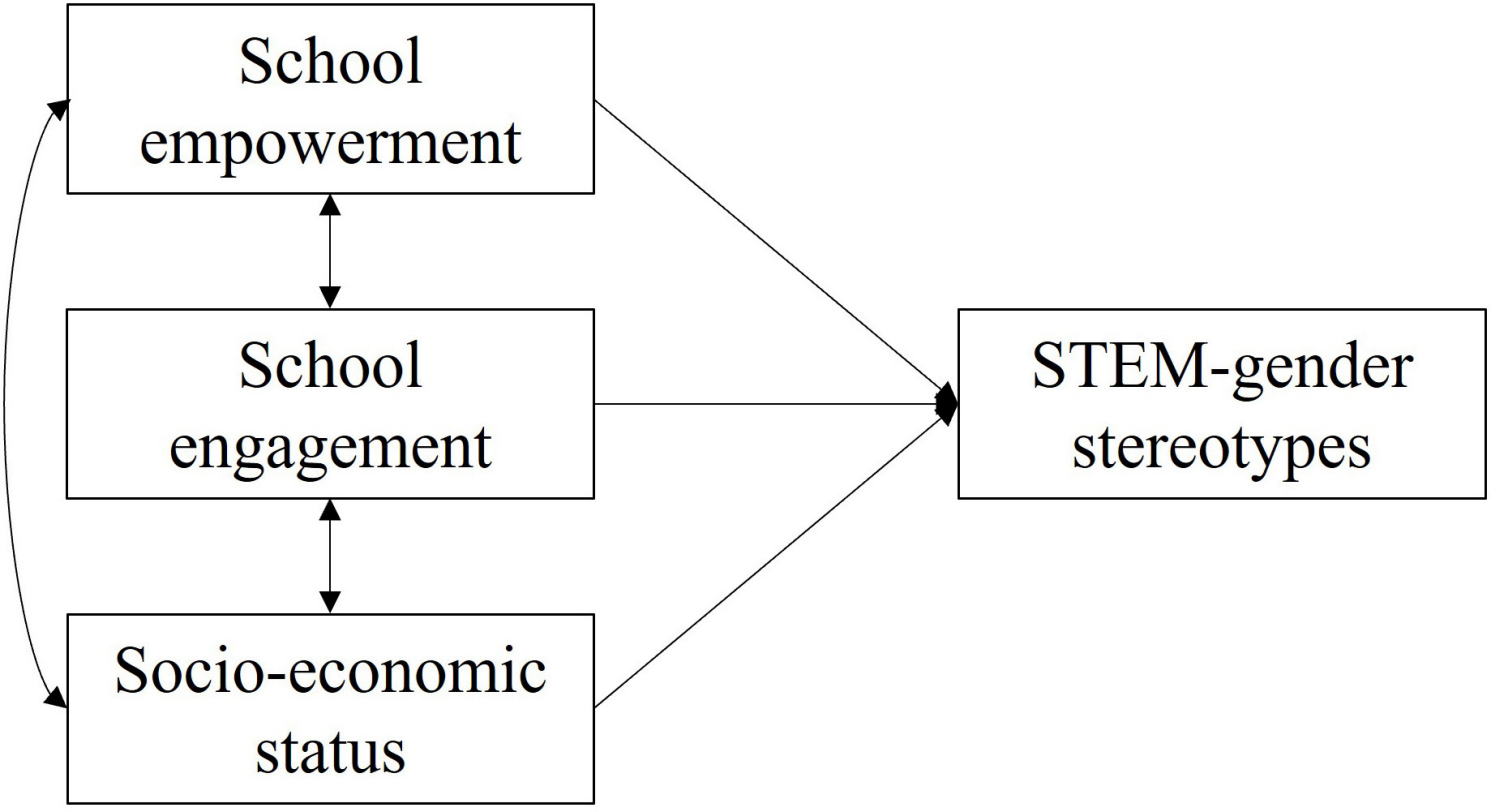
The theoretical model to be estimated across gender and national context (Italian males, Italian females, Nigerian males, and Nigerian females).

**Figure 2:**
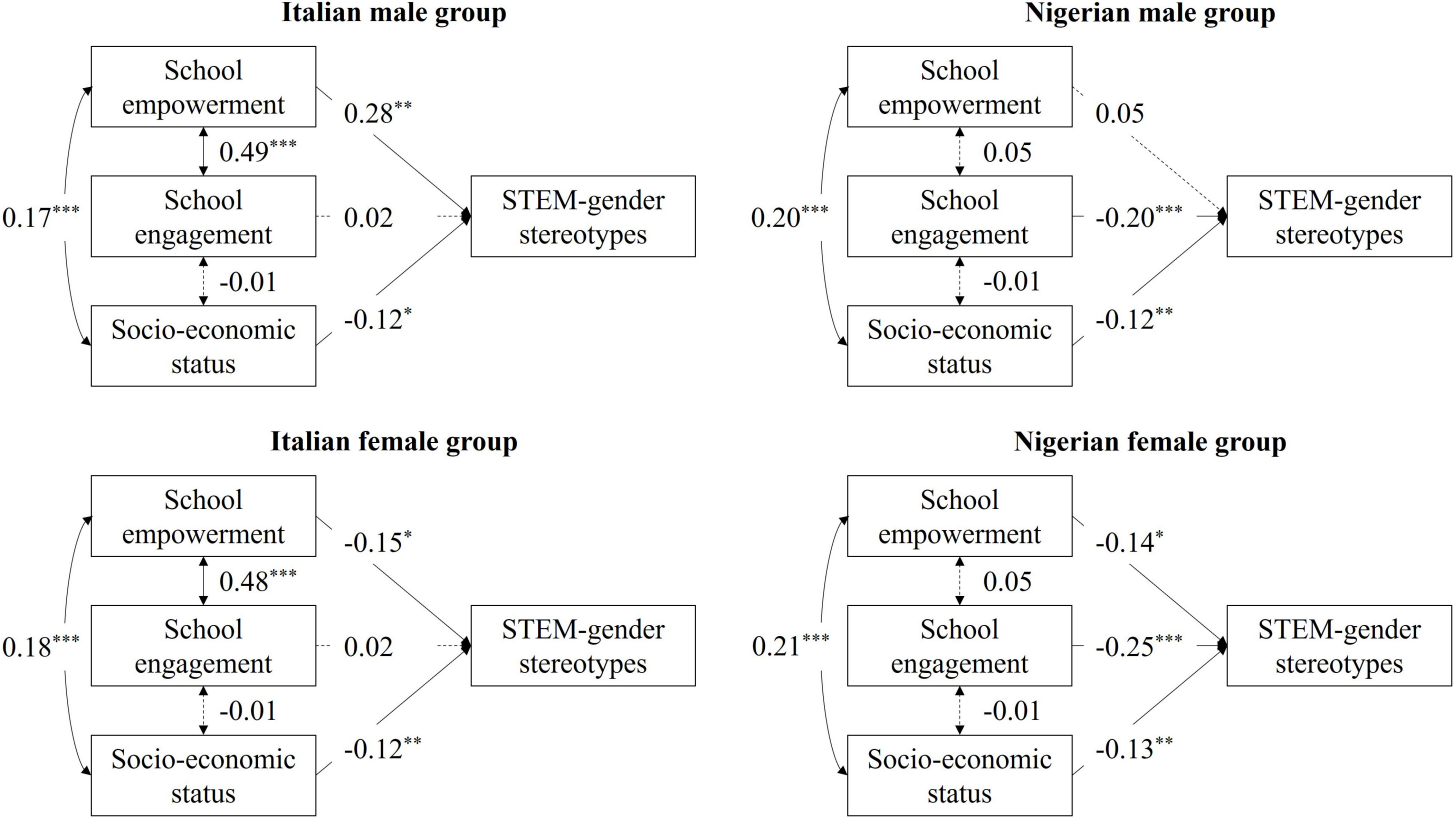
Final estimated multiple-group path model. Solid lines represent significant pathways, dashed lines are non-significant. Standardized regression coefficients (betas) are shown. *p < 0.05, **p < 0.01, ***p < 0.001.

The theoretical model to be estimated across gender and national context is illustrated in Figure 1. The initial unconstrained model was a saturated model, χ^2^(0) = 0.00, *p* = 0.00, CFI = 1.00, RMSEA = 0.000, SRMR = 0.000. The constrained version of the model had poor fit, χ^2^(18) = 74.62, *p* < 0.001, CFI = 0.000, RMSEA = 0.172, SRMR = 0.175 and a significantly worse fit compared to the unconstrained model, Δχ^2^(18) = 74.62, *p* < 0.001, ΔCFI = −1.00, ΔRMSEA = 0.172, ΔSRMR = 0.175. Inspection of modification indices suggested releasing the constraints for (a) paths from school empowerment to STEM-gender stereotypes in the Italian male and Nigerian male groups, (b) paths from school engagement to STEM-gender stereotypes in the Italian groups compared to the Nigerian groups, and (c) covariances between school empowerment and school engagement in the Italian groups compared to the Nigerian groups. The obtained partially constrained model had excellent fit, χ^2^(14) = 6.84, *p* = 0.94, CFI = 1.00, RMSEA = 0.000, SRMR = 0.036 and did not have a significantly different fit compared to unconstrained model, Δχ^2^(14) = 6.84, *p* = 0*.9*4, ΔCFI = 0.000, ΔRMSEA = 0.000, ΔSRMR = 0.036. Standardized coefficients of this final model are shown in Figure 2.

School empowerment was significantly and negatively related to STEM-gender stereotypes in both Italian and Nigerian female groups, while this association was significantly positive in the Italian male group and no significant relation was evidenced for the Nigerian male group. School engagement was significantly and negatively associated with STEM-gender stereotypes only in the Nigerian groups, while no significant relations were present in the Italian groups. SES was significantly and negatively linked to STEM-gender stereotypes in all considered groups. Furthermore, SES and school empowerment were significantly and positively correlated in all groups, while school empowerment and school engagement were significantly and positively associated only in the Italian groups and no significant relations were found in the Nigerian groups. No significant associations were revealed between SES and school engagement in all groups.

## Discussion

The purpose of the study was 2-fold. First, it investigated differences by gender and national context (Italy vs. Nigeria) in adolescents' STEM-gender stereotypes, school empowerment, and school engagement. Second, and more importantly, for the first time, it simultaneously analyzed how adolescents' STEM-gender stereotypes are related to the individual resources of school empowerment and school engagement as well as to the social factor of SES. These relations were considered in the context of the potential moderating role of gender and national context. The main results revealed that boys outscored girls in STEM-gender stereotypes and school empowerment and that Nigerian adolescents outperformed the Italian adolescents in STEM-gender stereotypes, school empowerment, and school engagement. Furthermore, higher school empowerment was significantly associated with lower STEM-gender stereotypes for girls regardless of the national context, while higher school engagement was associated with lower STEM-gender stereotypes in the Nigerian groups. Higher SES was associated with lower levels of STEM-gender stereotypes regardless of gender and national context. These results might suggest that, in addition to SES, school empowerment and school engagement can be relevant to be studied and to develop strategies to counteract STEM-gender stereotypes in adolescence. Nonetheless, it is necessary to consider the role of gender and national context to provide a better and appropriate interpretation of the emerging dynamics.

### Gender and Cultural Differences in Adolescents' STEM-Gender Stereotypes, School Empowerment, and School Engagement

We expected higher levels of STEM-gender stereotypes for boys than girls and for the Nigerian than the Italian contexts. Our findings supported this prediction. As previous literature extensively reported (e.g., Moè et al., 2021; Starr and Simpkins, 2021), in adolescence, boys endorse STEM-gender stereotypes more strongly than girls. One explanation for this is that generally people conform more easily to associations that favor their gender. STEM-gender stereotypes propose associations that favor boys (e.g., “STEM = male”), while disadvantaging girls (“girls have less ability than boys”). This process of “favoritism” could, in turn, increase the perception of congruity between boys' gender role (what choices and behaviors they consider typical for their gender) and the beliefs that members of society usually have about what is most appropriate for them (Eagly and Karau, 2002), further fostering their stereotypes. Favoritism and gender role congruence, therefore, may account for the higher levels of STEM-gender stereotypes in boys than girls.

The national context, along with related cultural and social features, is also a principal factor that differentiates the levels of STEM-gender stereotypes. In line with prior research (UNICEF, 2020b; World Economic Forum, 2021), we found much higher levels of STEM-gender stereotypes in Nigeria than in Italy. Nigeria is a low-middle-income country with a high gender gap. It is still facing serious issues regarding gender differences (Salman et al., 2011; Abdullahi et al., 2019), linked to religious dimensions, cultural traditions (e.g., early marriages for girls), and socio-political issues (e.g., the general education levels of the population). In this context, STEM-gender stereotypes are widespread in the Nigerian population, resulting in a greater inequality of opportunities between boys and girls. Looking at the 2021 GGGP data (World Economic Forum, 2021), these sociocultural processes seem less relevant in Italy, where boys and girls can have a more equal view of future life and professional chances. This could explain why Italian boys and girls have lower levels of STEM-gender stereotypes.

We also expected higher levels of school engagement for girls than boys as well as for the Nigerian than the Italian adolescents. The findings supported this prediction only partially. First, as hypothesized, our adolescent participants in Nigeria showed higher levels of school engagement. When a context generally offers fewer prospects for personal life and profession, as in Nigeria than in Italy, the school may be perceived as one of the most significant and catalyzing environments providing opportunities for social climbing. This can lead to living in school in a more active and energetic way, building more positive attitudes toward the academic experience, fueling greater concentration in achieving goals, and ensuring more meaningful social relationships (Furrer and Skinner, 2002; Salmela-Aro and Upadyaya, 2012). Second, our expectation of higher levels of school engagement for girls than boys was not supported, and no differences were found. This is not consistent with previous research, which suggested that personality and motivation factors (e.g., Lam et al., 2012) or differential expectations of parents and teachers (Wilkinson and Marrett, 1985; Eccles, 2007; Wang and Eccles, 2012) may promote girls' greater concern on their school connection and performance. Probably, this result should be interpreted in relation to our group of participants and the period of data collection. As mentioned above, all our participants attended the last year of lower secondary school in the last period of the school year, when shortly thereafter they would face the final exams and the choice of the higher education path. This may have favored a general greater engagement by all students toward the final goal, flattening any inter-individual and gender differences. Therefore, further studies with larger samples, at different school grades, and at various times of the school year would be desirable, especially if the design is longitudinal.

We took an exploratory approach in considering gender and cultural differences in mean levels of school empowerment. Boys and Nigerian participants showed higher school empowerment than girls and Italian participants. In terms of gender differences, a previous study showed significantly higher mean scores of girls than boys for school empowerment, but the effect size was small (Årdal et al., 2018). However, the school empowerment measure was not strictly comparable to that of our study. Årdal et al. (2018) used a measure referring to motivation for influencing school, perceived control, and participatory behavior (Ozer and Schotland, 2011). Our measure was related to meaning, competence, self-determination, and impact. The main difference can be identified in the inclusion, in our measure, of the competence dimension, for which boys usually score higher than girls (e.g., Conway et al., 2015; Gomez-Baya et al., 2019). This can at least partially explain our result. Nevertheless, this finding raises the question of whether social norms and cultural stereotypes can have a strong impact on girls, inhibiting those empowerment and assertiveness skills crucial for the promotion of their interests and demands (Hentschel et al., 2019). This topic should be addressed in future research. Regarding the higher levels of school empowerment of Nigerian students compared to Italian ones, this again seems to support the idea that in Nigeria, more than in Italy, school seems to be a significant and catalyzing context for the expression of the individual resources of boys and girls, who seem to be better able to experience school as a setting of active responsibility.

### Associations of Adolescents' STEM-Gender Stereotypes With School Empowerment, School Engagement, and SES in the Context of the Moderating Role of Gender and National Context

Concerning our primary goal, the findings showed that our expectations were generally supported with some exceptions. As expected, higher school empowerment was associated with lower levels of STEM-gender stereotypes in the two groups of girls regardless of the national context. Higher levels of school empowerment contribute to giving girls more control over their lives (Page and Czuba, 1999), by focusing on personal goals and enhancing their power in social interaction (Cattaneo and Chapman, 2010). This can make it easier for girls to react to the socially widespread STEM-gender stereotypes, which in contrast reduce their active self-determination and participation. The dynamic characterizing the boys is different. No significant association between school empowerment and STEM-gender stereotypes was evidenced for the Nigerian boys, while the association was positive for the Italian boys. Given that boys belong to the “gender favored by STEM-gender stereotypes,” it is not relevant for them to refer to empowering processes to counteract their social beliefs. On the contrary, the active management of social power might favor increasing levels of STEM-gender stereotypes. In line with this argument, it makes sense to expect this second mechanism to emerge in national contexts with a greater rate of individuality and where personal goals and success take on high relevance, such as in Italy, rather than in more collectivist contexts, such as Nigeria (Hofstede, 2001).

As far as the relations between school engagement and STEM-gender stereotypes are concerned, we found significantly negative links in the two groups of Nigerian boys and girls, while no associations were evidenced in the Italian groups. We hypothesized these differences related to the context, but we also expected some differences concerning gender. More specifically, we assumed a negative link between school engagement and STEM-gender stereotypes in the Italian girls, albeit less strong than that of the Nigerian girls. When students feel particularly engaged with the school, they are more inclined to consider it as a source of personal improvement, support, and motivation (Salmela-Aro and Upadyaya, 2012). This expands one's enthusiasm and interest also toward fields that social stereotypes would suggest as unsuitable and motivates to oppose these stereotypes. Such a process could therefore explain how school engagement would help reduce STEM-gender stereotypes in the Nigerian girls. However, this process might interact with the national context of reference. The more the school is considered a social value in terms of opportunities for social mobility in low-middle-income contexts, such as Nigeria, the more this process could be relevant and unfold its effects. In contexts where the levels of economic development and social support are higher, such as in Italy, the school could instead be perceived as a less determining factor for future subsistence, and this could dampen the fundamental meaning of the hypothesized process. This could be one reason for the lack of significant relation between school engagement and STEM-gender stereotypes in Italian girls. The two Italian and Nigerian contexts probably differ in another aspect as well. In the Nigerian context, where greater gender gaps and STEM-gender stereotypes are present, greater school engagement may imply greater attention to information counteracting these issues at school, and to girls when they present clear STEM skills. This could explain why school engagement was negatively associated for Nigerian boys in an equally relevant way as for Nigerian girls, while no significant relation was found for Italian boys, living in a context characterized by significantly lower levels of gender gap and STEM-gender stereotypes than in Nigeria (and this may make boys generally less sensitive to information and experiences promoting gender equity at school).

Finally, higher SES is related to lower levels of STEM-gender stereotypes, regardless of gender and national context. We expected this finding to be particularly relevant for girls compared to boys. In fact, we thought that higher levels of SES provided better chances for STEM learning and skills. Such a situation could more easily lead girls to reduce STEM-gender stereotypes than boys. However, our results suggest that SES background is equally relevant for boys as well. Although the literature suggests that STEM-gender stereotypes are more prevalent among adolescents than younger children due to advanced cognitive abilities connecting their identity with social categories (e.g., Passolunghi et al., 2014; Miller et al., 2018; Starr and Simpkins, 2021), however, other changes related to critical and moral skills could be generally associated with lower levels of STEM-gender stereotypes (e.g., Malti et al., 2021). A higher SES background could foster such skills for both girls and boys, and this could more easily explain our findings showing a lack of gender differences.

### Limitations, Strengths, and Future Research

This study should be considered in light of some weaknesses. First, we used a convenience sampling method to collect our research data, and this casts doubt on the generalizability of our results. Also, because of selection bias, it is possible that the schools that participated in the study were significantly more motivated and/or more satisfied with their education paths and activities than those which did not. Large population-based random samples would be ideal to be considered in future research. Second, the use of self-report measures requires caution when interpreting the findings, even more when diverse cultural contexts are considered. Next investigations should combine mixed methods. For example, the simultaneous use of qualitative and quantitative analysis could help highlight the subjective experience of boys and girls in various national contexts. Third, the cross-sectional nature of the study design precludes us from clearly concluding the direction of the associations among the study variables (for example, from school empowerment to STEM-gender stereotypes or vice versa). Thus, it would be important to conduct future longitudinal studies following the same participants during adolescence in order to draw clearer conclusions about the direction of associations between these variables and about the causality processes involved. Fourth, our study was limited to the investigation of the associations of school empowerment, school engagement, and SES with STEM-gender stereotypes within the context of potential differences by gender and nationality. Actually, other variables may be interesting to consider. For instance, further studies could consider how the family environment and parenting, peer experiences, teacher-student relationships, sense of community, and personal future expectations could directly or indirectly affect STEM-gender stereotypes (e.g., Tandrayen-Ragoobur and Gokulsing, 2021). Furthermore, it is noteworthy to point out that we focused on explicit stereotypes only, namely on conscious representations assessed through self-reports, which may produce biased responses due to social desirability. To prevent such concerns, many studies analyzed the role of implicit gender stereotypes in STEM performance (e.g., Hausmann, 2014). This suggests that future research should consider assessing both implicit and explicit stereotypes and comparing the results.

Despite these limitations, our study contributed meaningfully to the literature because it extends our understanding of the characteristic of STEM-gender stereotypes in two ways. First, it provided a new clear picture of how STEM-gender stereotypes may differ based on gender and nationality. Second, it revealed how significant and school-based variables (empowerment and engagement) are associated with STEM-gender stereotypes, considering the role of gender and national contexts in these relations. Together, the findings highlighted potential factors to work on to reduce STEM-gender stereotypes from an international perspective. However, interventions should be developed by taking into account gender and national differences.

### Educational Implication

Our findings provide implications for practice in the school community. Based on the STEMO model, we considered STEM-gender stereotypes as composed by two dimensions, i.e., cultural fit and ability stereotypes. To reduce the impact of these two types of stereotypes, it is important, on the one hand, to think of interventions that broaden the idea of who can be part of the STEM field and, on the other, to counteract the idea that skills are fixed (Master and Meltzoff, 2020). In the first case, for example, one could work by making the school environments dedicated to STEM teaching (for example, the computer room or the chemistry lab) less stereotypically masculine, using expedients such as the presence of plants, furniture with fluid lines, and colors usually matched to the feminine style (for example, powder pink and lilac). In the second case, it would be important for teachers to convey the idea that STEM skills are like a sporting activity: the more you practice and train, the more results will be obtained. Emphasizing the initial mistakes and failures of great scientists, who then achieved success by working hard, can be a good strategy. Moreover, in this line, Law et al. (2021) reported a good example of a growth mindset activity in a science museum. Both interventions seem particularly crucial to practice in contexts with high levels of stereotypes, such as Nigeria. Furthermore, they should be systematically addressed not only to girls, but also to boys, who are the holders of the highest levels of STEM-gender stereotypes and, therefore, as future fathers or managers, could hinder the STEM interests or careers of girls and women.

Based on our findings, it would also be important to design interventions to boost girls' school empowerment. To achieve this goal, schools and teachers should be committed to providing them with meaningful school environments, feelings of confidence in school work, opportunities for self-determination, and a sense of impact at school. Motivation training, aimed at making girls more confident and perceiving themselves as more able and capable to increase their performance, have proved to be effective interventions and deserve to be replicated (e.g., Moè, 2016). Another important strategy could be to provide positive role models in the use of empowerment skills (Master and Meltzoff, 2016). Teachers might represent such positive role models (they do not necessarily have to be females, just relatable and similar to the self along certain key dimensions), but also schoolmates who “are like me and manage to be influential and achieve their goals” can fulfill a similar function. Furthermore, simple activities such as assigning responsibility for leading teamwork could prove effective and easily applicable.

Intervention programs should also promote school engagement, being aware that such interventions are likely to be most effective where the value of the school is generally believed to be more socially crucial, such as in Nigeria compared to Italy. One way to achieve this goal is to strengthen the sense of belonging to the school, by proposing activities that reinforce the idea that school is a “meaningful context of life.” In this line, the redefinition of academic programs toward topics close to students' experiences, the offer of extracurricular activities of interest to them (concerning, for example, sports and music), the support of significant peer tutors, and motivational programs could be important.

It should be noted that all the interventions previously outlined involve the school microsystem of girls and boys. However, our study suggests that other factors related to the socioeconomic and cultural development of home nations and families also potentially play a role in the formation of STEM-gender stereotypes. At this level, economic and social policy interventions are desirable in the direction of providing more girls and boys with opportunities for knowledge and experiences in STEM. Such occasions should suggest that the involvement of both genders in the STEM field is a crucial point for the wellbeing and progress of our living communities.

## Data Availability Statement

The raw data supporting the conclusions of this article will be made available upon reasonable request by the authors, without undue reservation.

## Ethics Statement

The study was approved on 11 May 2020, by the Ethical Committee at the Department of Education, Psychology, and Communication at the University of Bari (Ethics reference code: ET-20-06). Written informed consent to participate in this study was provided by the participants' legal guardian/next of kin.

## Author Contributions

PM provided the conception and design of this study, performed and interpreted the data analyses, and wrote the first draft of the manuscript. ML and EI contributed to the conception of this study, collected data, drafted, and revised the article substantially. SA and CS revised the article and proposed important suggestions for modification. RC revised the article critically for important intellectual content. All authors contributed to the article and approved the submitted version.

## Conflict of Interest

The authors declare that the research was conducted in the absence of any commercial or financial relationships that could be construed as a potential conflict of interest.

## Publisher's Note

All claims expressed in this article are solely those of the authors and do not necessarily represent those of their affiliated organizations, or those of the publisher, the editors and the reviewers. Any product that may be evaluated in this article, or claim that may be made by its manufacturer, is not guaranteed or endorsed by the publisher.
